# Cellular Immune Responses in HIV-Negative Immunodeficiency with Anti-Interferon-γ Antibodies and Opportunistic Intracellular Microorganisms

**DOI:** 10.1371/journal.pone.0110276

**Published:** 2014-10-20

**Authors:** Jiraprapa Wipasa, Panuwat Wongkulab, Kriangkrai Chawansuntati, Romanee Chaiwarit, Khuanchai Supparatpinyo

**Affiliations:** 1 Research Institute for Health Sciences, Chiang Mai University, Chiang Mai, Thailand; 2 Department of Medicine, Chiang Mai University, Chiang Mai, Thailand; Federal University of São Paulo, Brazil

## Abstract

**Background:**

Cell-mediated immunity plays a crucial role in resistance to intracellular infection. We previously reported antibodies against interferon-gamma (IFN-γ) in HIV− negative (HIV−) patients with acquired immunodeficiency presenting with repeated episodes of disseminated infection caused by uncommon opportunistic intracellular fungal, bacterial, and viral pathogens. This follow-up study aimed to investigate cellular immune responses in these unusual patients.

**Methods:**

Twenty HIV− patients presenting with ≥2 episodes of culture- or histopathologic-proven opportunistic infections were enrolled along with age- and sex-matched controls comprised of 20 HIV+ patients plus 20 healthy adults. Monocyte phenotyping and intracellular cytokine production were determined by staining with specific antibodies followed by flow cytometry. Anti-interferon-γ antibodies were measured by enzyme-linked immunosorbent assay, and inducible nitric oxide synthase by reverse-transcription polymerase chain reaction.

**Results:**

There were no differences among cases, HIV+, and healthy controls in the percentage of monocytes, or CD68 and HLA-DR expression on their surfaces. FcR1 (CD119) expression on monocytes was significantly higher in cases than in HIV+ (*p*<0.05) and healthy controls (*p*<0.01), suggesting the presence of activated monocytes in the circulation. Interleukin (IL)-2 and tumor necrosis factor (TNF)-α production in CD4 cells were significantly lower in cases than in healthy controls (*p*<0.01 and *p*<0.001, respectively). CD8 production of TNF-α among cases was significantly lower than that of healthy controls (*p*<0.05).

**Conclusion:**

Immunodeficiency in HIV− individuals with repeated infections with intracellular pathogens may be associated with one or more of the abnormal immune responses reflected by the reduced production of both IL-2 by CD4 T cells and TNF-α by CD4 T cells and CD8 T cells, as well as presence of anti-IFN-γ antibody, as previously reported.

## Introduction

Cell-mediated immunity (CMI) plays a crucial role in resistance to intracellular infection [1], [2]. Activation of phagocytes, mainly macrophages and dendritic cells, results in the production of interleukin (IL)-12 and tumor necrosis factor (TNF)-α. IL-12 is a potent activator of interferon (IFN)-γ production from NK cells and from antigen-specific T-helper type 1 (Th1) cells. IFN-γ acts in synergy with TNF-α to enhance expression of the major histocompatibility complex (MHC) and co-stimulatory molecules, and to activate microbicidal substances such as reactive oxygen species, nitric oxide, and lysosomal enzymes of macrophages. With adequate help from CD4 T cells, aided by CD40-CD40L interactions and cytokine production, CD8 T cells become activated to mediate killing of virus-infected cells, producing IFN-γ and TNF-α to inhibit the pathogen replication [3]. IL-2 is also critical for proliferation of these effector cells. Successful orchestration of all these immune defense mechanisms usually leads to successful control of intracellular pathogens. Disruption of the immune system, however, either by primary immunodeficiency [1], [2] or by neutralization with antagonists, may lead to susceptibility to such infections, even by organisms that normally are weakly-pathogenic.

Reports continue to accumulate in recent years of adults with acquired immunodeficiency who are not infected with human immunodeficiency virus type 1 (HIV), but present with uncommon opportunistic intracellular microorganisms [4]–[9]. We also previously described 20 HIV-negative (HIV−) patients who presented with disseminated non-tuberculous mycobacterial (NTM) infection, disseminated penicilliosis marneffei, non-typhoidal *Salmonella* bacteremia, crytococcosis, histoplasmosis, and disseminated herpes zoster, with a relatively high 32% mortality by a median of 25 months after diagnosis [9]. Such patients did not receive immunosuppressive drugs, and had no identifiable underlying condition to explain their weakened immune systems [6], [7], [9]. Collective evidence suggests that anti- IFN - γ antibodies may be the cause of immunodeficiency in these patients [4]–[9]. However, other abnormalities in CMI cascades may also contribute to immunodeficiency in such patients and needs further study. We, therefore, investigated anti-IFN-γ antibodies and other possible defects in CMI to expand knowledge of the etiology of repeated infections with opportunistic intracellular infections in HIV− patients.

## Materials and Methods

### Study population

Among 109 patients diagnosed from 1991 to 2011 at the Chiang Mai University Hospital (Chiang Mai, Thailand) to have HIV-negative adult-onset immunodeficiency, a total of 20 recent patients were enrolled prospectively from March 2011 to March 2012 in a cross-sectional, case-control study, as reported previously [9]. They included 11 females and 9 males between the ages of 18 to 60 years. In brief, the most common opportunistic infections among the 20 were disseminated NTM infection (19, 95%), disseminated penicilliosis marneffei (12, 60%) and non-typhoidal *Salmonella* (7, 35%). Other unusual infections included cryptococcosis, histoplasmosis, and disseminated herpes zoster infection, each in one patient (5%). A high prevalence of anti-IFN-γ antibodies was found in these cases. Active cases were defined as those presenting with active opportunistic infections during the 30 days prior to study entry, and those requiring intravenous antimicrobials for these infections at that time. All cases were seronegative for HIV− 1 on the day of enrollment.

Based on the assumption that the prevalence of anti-IFN-γ antibodies among cases was 80% and in each of the control groups was 30%, sample-size calculations indicated a minimum of only 14 subjects in each study arm would be powered to show a significant difference with the type I error of 0.05 and the power of 80% between any two groups, if present. Thus, 20 age- and sex-matched HIV-infected (HIV+) controls and 20 similarly-matched healthy controls were enrolled for comparison. All healthy controls were HIV-negative at the time of enrollment. The absolute CD4 T cell counts were not different between cases and healthy controls, but HIV+ controls had significantly lower CD4 T cell counts than the other two groups [9] (Table 1). All HIV+ individuals were receiving highly active antiretroviral therapy.

**Table 1:** Demographic and clinical characteristics of cases of HIV-negative (HIV−) adult-onset immunodeficiency versus HIV-infected (HIV+) and healthy controls (from reference [9]).

Characteristic	HIV− casesN = 20^a^	HIV+ controls		Healthy controls^a^	
		N = 20^a^	*p* value^b^	N = 20^a^	*p* value^c^
Mean age (years) ± SD	48.1±6.4	48.3±6.3	1.000	47.1±6.5	0.627
Female sex − no. (%)	11 (55)	11 (55)	1.000	11 (55)	1.000
Mean body weight (kg) ± SD	52.3±11.6	56.5±11.4	0.118	59.9±9.4	***0.027***
**Laboratory findings**					
Hemoglobin (g/dL)	11.07±2.36	13.40±1.23	***<0.001***	13.51±1.88	***<0.001***
White blood cells (x1000/mm^3^)	12.6 (6.9, 19.2)	6.1 (4.7, 7.0)	***<0.001***	6.3 (5.2, 7.4)	***<0.001***
Absolute neutrophils (x1000 cells/mm^3^)	7.7 (4.0, 12.5)	3.1 (2.5, 4.1)	***<0.001***	3.5 (2.8, 3.9)	***<0.001***
Absolute lymphocytes (x1000 cells/mm^3^)	2.1 (1.7, 3.1)	1.9 (1.6, 2.2)	0.417	2.0 (1.8, 2.3)	0.715
Absolute eosinophils (x100 cells/mm^3^)	6.31 (4.15,10.31)	1.22 (0.85,3.41)	***<0.001***	1.78 (1.15,3.34)	***<0.001***
Absolute CD4+ count (cells/mm^3^)	662 (418, 890)	435 (346, 550)	***0.023***	692 (624, 834)	0.417
Absolute CD8+ count (cells/mm^3^)	609 (460,766)	775 (589,856)	***0.040***	523 (429, 611)	0.160
**Anti-IFN-γ antibodies**					
Prevalence	100%	0%		0%	
Mean assay OD^d^ at 492 nm ± SD	2.460±1.309	0.058±0.004	***<0.001***	0.059±0.005	***<0.001***
**Opportunistic infections**					
Non-tuberculous mycobacteria	20	0		0	
* Penicilliosis marneffei*	12	0		0	
Non-typhoidal salmonellosis	8	0		0	
Cryptococcosis	1	0		0	
Histoplasmosis	1	1		0	
* Herpes zoster*	1	2		0	
Cerebral toxoplasmosis	0	1		0	
* Pneumocystis jirovecii*	0	7		0	
Cytomegalovirus	0	2		0	
Tuberculosis	0	2		0	

a Data are expressed as the numbers of subjects, with percentage in parentheses (%), or as central tendency as means plus or minus (±) standard deviation in parentheses.

b Comparison between cases and HIV+ controls. Boldfacing indicates statistical significance.

c Comparison between cases and healthy controls. Boldfacing indicates statistical significance.

d OD = Optical Density.

The study was approved by ethics committees of the Faculty of Medicine and the Research Institute for Health Sciences of Chiang Mai University. Written informed consent was obtained prior to enrolment.

### Determination of anti-IFN-γ antibody

Anti-IFN-γ antibody in sera was determined by indirect enzyme-linked immunosorbent assay (ELISA), as previously detailed [9]. Briefly, Maxisorb immunoplates (Nalge Nunc International, Penfield, NY, USA) were coated with 1 µg/mL of recombinant human IFN-γ in bicarbonate buffer (pH 9.6) and kept overnight at 4°C. Plates were blocked with phosphate-buffered saline (PBS) containing 1% non-fat milk powder at 37°C for 1 hour. Sera diluted 1∶1,000 were incubated for 1 hour at 37°C in duplicate. Plates were subsequently developed with anti-human IgG horseradish peroxidase conjugate (Caltag Laboratories, Invitrogen Life Technologies, Thermo Fisher Scientific, Paisley, UK) followed by *o*-phenylenediamine substrate (Sigma-Aldrich, St. Louis, MO, USA). The enzyme reaction was terminated with sulfuric acid (2N) and read for absorbance at 492 nm on a Spectra MR plate reader (Dynex Technologies, Chantilly, VA, USA).

### Preparation of peripheral blood mononuclear cells

Seventeen milliliters of venous blood were collected and peripheral blood mononuclear cells (PBMCs) were separated from citrated blood by gradient centrifugation over Ficoll-Hypaque (Amersham Biosciences, GE Healthcare, Little Chalfont, UK). Remaining erythrocytes were removed by incubation with lysis buffer (0.15 M NH4Cl, 10 mM KHCO3, 0.1 mM Na2EDTA) at room temperature for 5 minutes. The cells were washed twice with RPMI, resuspended in 10% fetal calf serum/RPMI culture medium (R10), and then stained with trypan blue to identify viable cells for counting and adjustment to the required concentration.

### Monocyte phenotyping by flow cytometry

To characterize monocytes, one million PBMCs in 1 mL R10 were cultured with 1 ng/mL IFN-γ or medium alone at 37°C, 5% CO_2_ for 24 hours. Cells were then stained with fluorochrome-conjugated anti-human CD14, anti-human CD68, anti-human human leukocyte antigen (HLA)-DR, and anti-human interferon γ receptor 1 (FcγR1, CD119) (all from Biolegend, San Diego, CA, USA) and then counted for monocyte populations and CD14+ cellsby flow cytometry (CyAn ADP analyzer, Beckman Coulter, Fullerton, CA, USA) using Summit software (Fullerton, CA, USA). Gates were set on monocyte population on forward scatter versus side scatter dot plot.

### Intracellular cytokine production

Assay to determine intracellular cytokine production was performed by our standard protocol described previously [10]. Briefly, one million PBMCs were stimulated with 5 µg/mL phytohemagglutinin (PHA) (Sigma-Aldrich) or medium alone at 37°C, 5% CO_2_ for 20 hours. Brefeldin A (Sigma-Aldrich) at a final concentration of 10 µg/mL was added to cultures for the last 4 hour of incubation. Each culture was divided into two aliquots, washed with FACS buffer, and stained with fluorochrome-conjugated anti-human CD3, anti-human CD4, anti-human CD8, anti-human CD69, and anti-human CD45RO for 30 min at 4°C. The cells were fixed with 4% paraformaldehyde (PFA), permeabilized with 0.2% saponin (Fluka, Sigma-Aldrich) in FACS buffer for 10 min at room temperature and incubated with anti-human IL-2, anti-human IL-10, anti-human IFN-γ, and anti-TNF-α for 30 min at 4°C. They were then washed and fixed in 1% PFA in PBS. Corresponding isotype controls were used. The cells were analysed by flow cytometry using forward and side scatter, for which 100,000 lymphocytes were collected.

### Inducible nitric oxide synthase production

To detect inducible nitric oxide synthase (iNOS), 1 million PBMCs in 1 mL R10 were stimulated by culture with 1 ng/mL IFN-γ or medium alone at 37°C for 24 hours. Cells were centrifuged and the pellet was stored in RNA stabilization reagent (RNAlater®, QIAGEN, Hilden, Germany) at −20°C until analysis. Total RNA was extracted using Ribozol RNA extraction reagent (Amresco, Solon, OH, USA) and subjected to semi-quantitative reverse transcriptase polymerase chain reaction (RT-PCR), modified from the method described previously [11]. Briefly, 10 ng of total RNA was reverse transcribed and RT-PCR was performed using the One-Step RT-PCR kit (QIAGEN) to detect iNOS. The primer used for sense was 5′ TGCAGACACGTGCGTTACTCC 3′, and for antisense was 5′ GGTAGCCAGCATAGCGGATG 3′. PCR reaction products were electrophoresed on 2% agarose gel, stained with ethidium bromide and quantified by UV transluminator image documentation system using GeneSnap program (Syngene, Cambridge, UK). The levels of PCR products specific to cytokines were normalized relative to glyceraldehydes 3-phosphate dehydrogenase (GAPDH) amplicon mRNA. Fold increase of iNOS mRNA expression when PBMCs were stimulated with IFN-γ was calculated relative to unstimulated controls.

### Statistical analysis

The Kruskal-wallis one-way analysis of variance by rank test was used for comparisons of immune parameters between cases and each group of controls. The Mann-Whitney U test analyzed differences in cytokine responses between patients with and without active opportunistic infection at the time of study entry. All analyses were performed using GraphPad Prism Software (La Jolla, CA, USA).

## Results

### Monocyte characteristics

**Figure 1:**
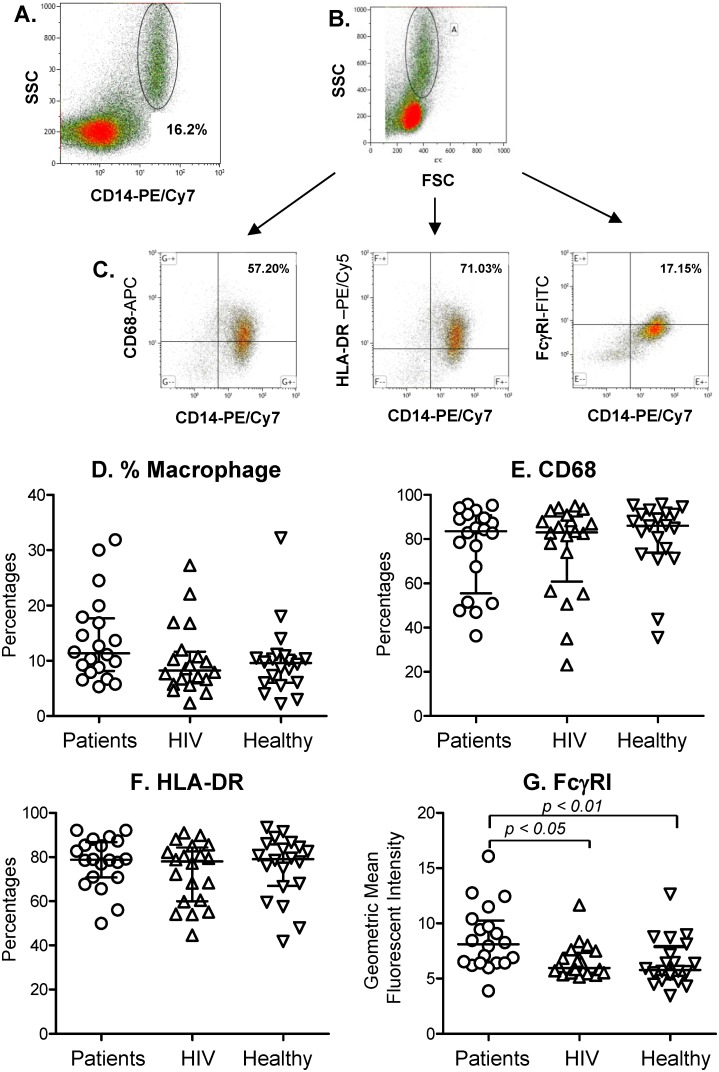
Flow cytometric characterization, frequencies, and function of macrophages. PBMCs were stained with fluorochrome-conjugated anti-CD14, CD68, HLA-DR, and FcγR1. Monocyte/macrophage populations were identified by gating forward, side scatter, and CD14+ (A). Gates were then set on monocyte population on forward scatter and side scatter dot plot (B) and analyzed for the expression of CD68, HLA-DR and FcγRI in relation to CD14 expression (C). Figures show the percentages of (D) macrophages overall, (E) macrophages expressing CD68, (F) macrophages expressing HLA-DR, as well as (G) the mean fluorescent intensity of FcγR1. Symbol represents the data of each individual case patient (circles), HIV+ control (up-pointing triangles), and healthy control (down-pointing triangles). Horizontal long lines show the medians, and short lines the interquartile ranges of each group. Indicated *p* values for comparisons are by Kruskal-wallis one-way analysis of variance by rank test.

We found no differences in the percentages of monocytes identified by expression of CD14 among cases, HIV-infected controls, nor healthy controls (Fig. 1D).

The percentages of CD14^+^ monocytes expressing CD68, a molecule involved in cell adhesion of macrophages to particular sites [12], and HLA-DR, did not differ among the three study groups (Fig. 1E and 1F). The geometric means of fluorescence intensity of HLA-DR on CD14^+^ monocytes were also not statistically different (data not shown). Among the cases, FcγRI expression on CD14^+^ monocytes was statistically significantly higher than in HIV+ (*p*<0.05) and healthy controls (*p*<0.01) (Fig. 1G), suggesting the presence of activated monocytes in the circulation of cases.

### Inducible nitric oxide synthase production

iNOS is essential for killing of intracellular pathogens. The average fold increases of iNOS production in response to IFN-γ were not different among groups, suggesting that the ability of PBMCs of cases to produce iNOS was normal (data not shown).

### Cytokine production

**Figure 2:**
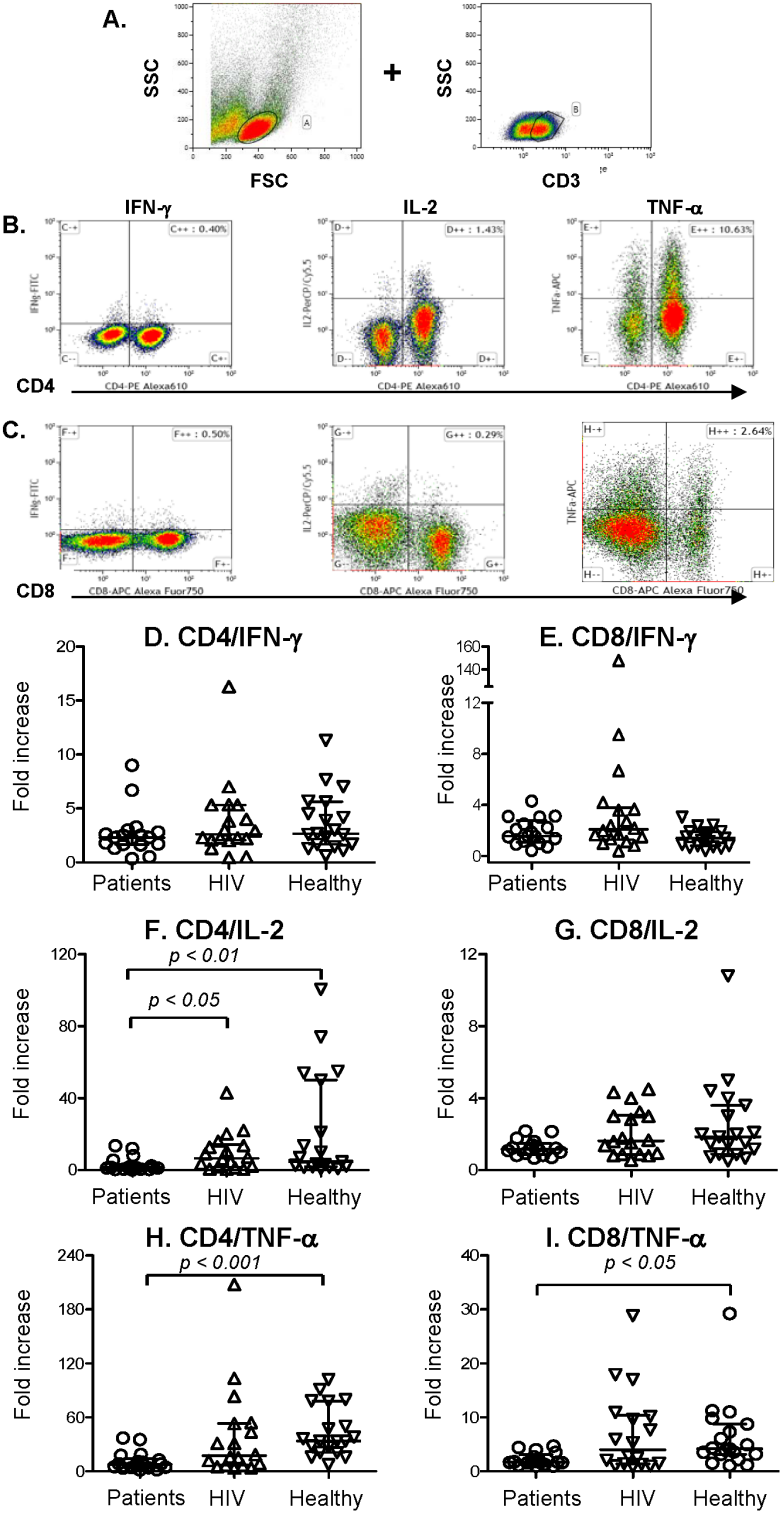
Cytokine production by CD4 and CD8 T cells, by CD4 and CD8 T-cell types, and by fold increase upon stimulation. PBMCs were stimulated by phytohemagglutinin or by no mitogen stimulant for 24 hours. Cells were harvested and stained with fluorochrome conjugated CD3, CD4, CD8, IFN-γ, IL-2 or TNF-α mAbs. PBMCs were gated by forward and side scatter and the CD3^+^ T lymphocyte population was identified (A). IFN-γ, IL-2 or TNF-α expression in CD4^+^ T cells (B) or CD8+ T cells (C) were then examined. Figures 2D and 2E show fold increase of IFN-γ, Figures 2F and 2G fold increases of IL-2, and Figures 2H and 2I fold increases of TNF-α production in response to PHA, compared to cells cultured with no mitogen stimulant. Symbols represents the data of each individual case patient (circles), HIV+ control (up-pointing triangles), and healthy control (down-pointing triangles). Long horizontal lines show the medians, and short lines the interquartile ranges of each group. Indicated *p* values for comparisons are by Kruskal-wallis one-way analysis of variance by rank test.

There were no statistically significant differences between cases and controls in fold increases for the percentages CD4 and CD8 T cells producing IFN-γ in response to PHA stimulation, compared to medium alone (Fig. 2D and 2E). IL-2 production by CD4 T cells of cases was significantly lower than that of HIV+ (*p*<0.05) and healthy controls (*p*<0.01) (Fig. 2F). The median fold increase of CD8 T cells producing IL-2 among cases was 1.17 (interquartile range, 0.88 to 1.49), which was lower than that of HIV+ controls (median, 1.63; interquartile range, 0.87 to 3.06) and healthy controls (median, 1.85; interquartile range, 0.89 to 3.60), however, these numbers did not differ statistically (Fig. 2G).

Cases produced significantly less TNF-α than healthy controls in their CD4 and CD8 T cells (*p*<0.001 and *p*<0.05, respectively) (Fig. 2 H and 2I). Among cases, the median fold increase of TNF-α production by CD4 T cells was 7.7 (interquartile range, 4.02 to 13.99) and by CD8 T cells was 1.7 (interquartile range 1.43 to 3.10, respectively). These were lower than that of HIV+ controls (CD4 median 17.2; interquartile range, 6.66 to 53.28; and CD8 median 4.02, interquartile range 1.40 to 10.38), but ANOVA did not reveal significant differences between the groups. Although HIV+ controls had significantly lower numbers of CD4 T cell counts than the other two groups [9], cytokine production by CD4 and CD8 T cells in response to PHA stimulation of the HIV+ did not differ from that of healthy controls.

**Figure 3:**
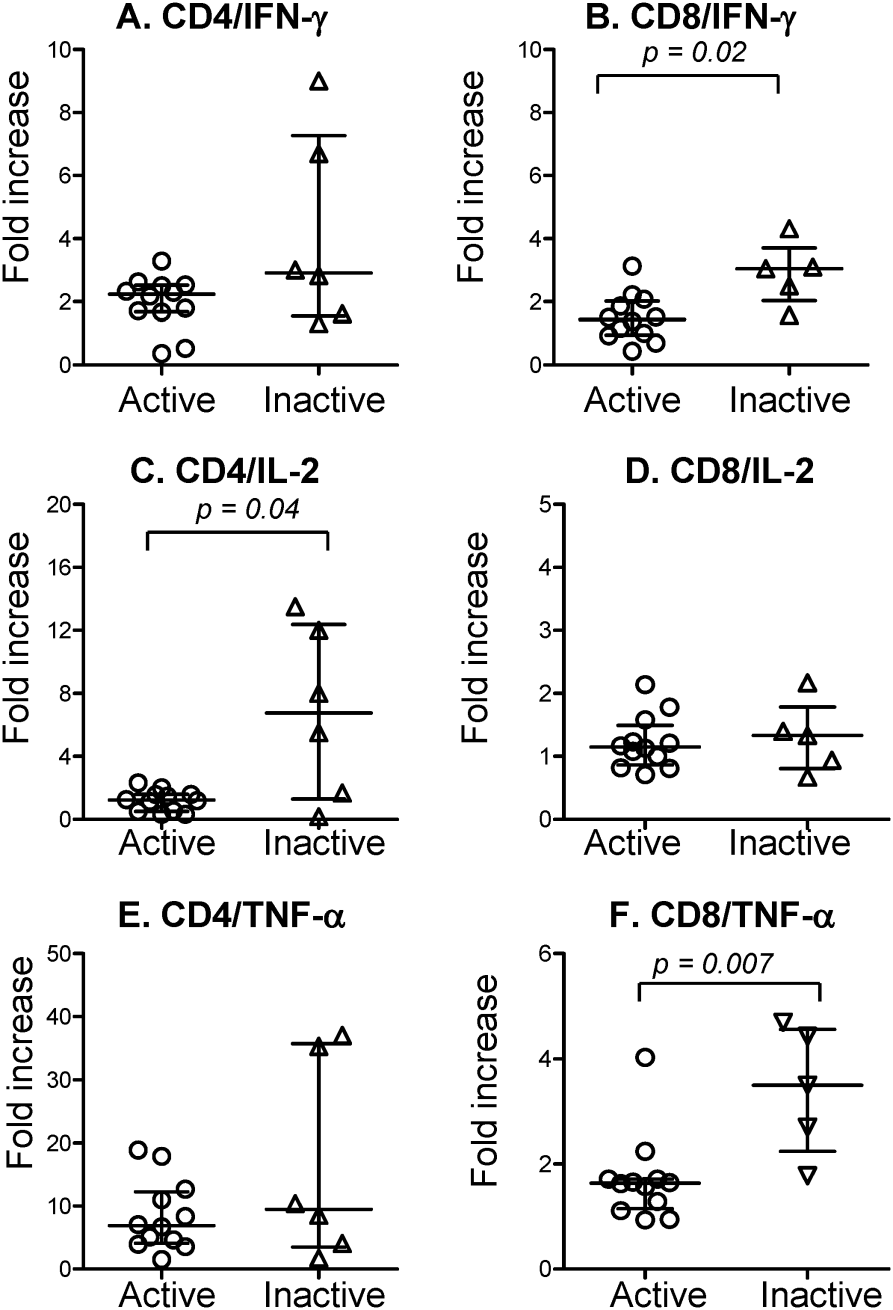
Comparison of cytokine production between active cases (circles) and inactive cases (triangles) with opportunistic infections. Solid lines show the medians with interquartile ranges of each group. Indicated *p* values for comparisons are by unpaired t-test.

CD8 T cells from active cases with opportunistic infections produced significantly less IFN-γ and TNF-α than that of inactive cases (*p* = 0.02 and *p* = 0.007, respectively) (Figure 3B and 3F). CD4 T cells producing IL-2 of active cases were also lower than that of inactive cases (*p* = 0.04) (Figure 3C).

## Discussion

Our observation that patients with active opportunistic infections had higher levels of antibody against IFN-γ compared to cases with “inactive” opportunistic infections is consistent with the hypothesis that IFN-γ autoantibody may be the cause of immunodeficiency in patients with unusual and often disseminated infections by intracellular microorganisms [4]–[9]. The biological action of this autoantibody is still unclear, although several studies suggest it may physiologically inhibit IFN-γ and its downstream responsive abilities [6], [8], [9], [13], [14]. Nevertheless, anti-cytokine antibodies can also be found in healthy individuals [15], and it has been postulated that the production of such antibodies may be a homeostatic balancing as a result of persistently elevated levels of a specific cytokine [16]. While the precise role of anti-IFN-γ antibody in patients infected with unusual intracellular microorganisms remains to be elucidated, perhaps other defects in CMI may also contribute to immunodeficiency in these patients.

We did not find differences in the percentages and characteristics of monocytes among cases, HIV− infected and healthy controls. Similarly, PBMCs from cases produced iNOS – which plays an important role in host defense – at the same level as controls. Diminished iNOS production has been shown to be associated with susceptibility to infections and disease severity in humans [17]–[20]. Although receptors for IFN-γ on monocytes were not assessed in this study, our finding of normal iNOS production after IFN-γ stimulation suggests that IFN-γ receptor were functional in the cases. Other studies of similar adult-onset immunodeficiency also reported the normal ability of monocytes to produce TNF-α [6], [21]. These collective data indicate that the functions and characteristics of monocytes of HIV− immunodeficiency patients are normal. The higher expression of FcγRI on CD14^+^ monocytes observed among our patients may simply reflect prolonged activation of their immune systems.

Only a few have studied the function of CD4 and CD8 T cells among patients infected with opportunistic intracellular microorganisms and also seropositive for anti-IFN-γ antibody [6], [8], [21]. Most of these reports assessed the overall function of PBMCs by the determination of cytokines in the supernatant after cell stimulation. In contrast, our study determined CD4 and CD8 cytokine production by direct staining with specific antibodies and flow cytometry, providing information on the frequency and phenotypes of cytokine-producing cells. Thus, we found in our cases reduced IL-2 production by CD4 T cells, and reduced TNF-α by both CD4 and CD8 T cells in response to mitogen stimulation. These reductions were more pronounced in cases with active versus inactive opportunistic infections.

IL-2, produced mainly by T-cells [22], is a potent cytokine which mediates proliferation and activities of various effector cells. Decreased IL-2 production in humans has been shown to be associated with susceptibility to infections such as cytomegalovirus [23], *Cryptococcus neoformans* [24], *Onchocerca volvulus* [25] and pulmonary tuberculosis [26]. Impaired IL-2 production in response to purified protein derivative of tuberculin has been reported in a group of patients infected with NTM in Japan [27]. A patient with *Mycobacterium avium* also presented diminished IL-2 production following PHA stimulation [28]. The precise consequence of IL-2 deficiency in these patients has not been investigated. Since IL-2 is a multifunctional cytokine, it is plausible that its deficiency disturbs proliferation and differentiation of effector cells, resulting in immune dysfunction.

TNF-α is produced primarily by macrophages and T cells [29], with a wide range of effects, including cell activation, proliferation, differentiation, survival, and cell death [30]. TNF-α plays a crucial role in intracellular infection by activating macrophages and by promoting migration of other immune cells to the site of infection. Blocking TNF-α by antagonists results in increased susceptibility to unusual pathogens. For example, patients with ulcerative colitis who were treated with an anti-TNF-α agent were prone to infection with *Listeria monocytogenes*, a bacterium that mainly affects immunocompromised hosts [31]. Rheumatoid arthritis patients receiving TNF-α blockers were reported to become infected with *Pneumocystis carinii* [32], *Legionella pneumophila* [33], [34], *M. tuberculosis* [35], *Echinococcus multilocularis* [36], *Histoplasma capsulatum* [37], non-typhi Salmonella [38], [39], and non-tuberculous mycobacteria [40]–[43]. These data indicate the importance of TNF-α in controlling intracellular organisms. A significant reduction of TNF-α production by CD4 and CD8 T cells of the cases in our study may have contributed to their susceptibility to unusual intracellular pathogens.

It also remains possible that the reduction of cytokine responses in our cases might be a consequence of T cell exhaustion following chronic antigenic stimulation [44], [45]. This phenomenon is characterized by a progressive impairment of effector function, decreased cytokine production, inability to elaborate the usual array of effector activities, and the expression of inhibitory receptors [45], [46]. The diminished IL-2 and TNF-α production by T cells observed in our study is consistent with previous reports finding that in chronic infections IL-2 production, and then TNF-α production, were the first effector activities to be interrupted, while IFN-γ was more resistance to functional exhaustion [45], [47]–[49]. Cytokine production and cell proliferation may be partially restored if the antigen load can be brought under control [50]. Due to limitations in the cells we could obtain, T-cell exhaustion was not investigated in this study. Longitudinal data collection on phenotypic and functional changes may in future clarify whether defective cytokine production can be restored after patients are cured from their infections.

It is unclear whether the immunodeficiency in patients observed in our study results solely from the production of anti-IFN-γ antibodies or it is worsening by the reduction of IL-2 and TNF-α production. An apparent association of autoantibody and decreased cytokine production has been clearly demonstrated in systemic lupus erythematosus (SLE) patients. Anti-T cell receptor (TCR)/CD3 antibodies in sera of SLE patients activate Ca(2+)/calmodulin-dependent kinase IV (CaMKIV) and induce binding of a transcriptional inhibitor of IL-2 gene promoter, resulting in the suppression of IL-2 production [51]. Decreased IL-2 production in SLE patients contributes to multiple defects of host immunity [52]. The association of autoantibody and the reduction of TNF-α has been less well established. Understanding a relation of immunological abnormalities will clearly provide insights into potential management of the disorder. Further analysis of the relative importance of the presence of anti-IFN-γ antibodies and decreased IL-2 and TNF-α production in HIV-negative immunodeficiency with anti-interferon-γ antibodies and opportunistic intracellular microorganisms is warranted.

In conclusion, we demonstrated that patients infected with opportunistic intracellular microorganisms have normal monocytes percentages and function. Their reduced IL-2 produced by CD4 T cells, and their reduced TNF-α by both CD4 and CD8 cells may explain the immunodeficiency of such HIV− patients, in addition to the finding of autoantibodies to IFN-γ. However, our measurement of cytokine production was by non-specific mitogenic stimulation. Further investigation of cytokine responses stimulated by specific antigens is thus warranted. Long-term studies of patients recovering from this phenomenon of unexplained, HIV−, adult-onset immunodeficiency may provide a better understanding of its etiologic mechanisms.
